# Drag Expression Among Older Gay Men: Exploratory Findings of a Pilot Study

**DOI:** 10.1093/geroni/igab046.3589

**Published:** 2021-12-17

**Authors:** Brian Chapman, Laura Donorfio, Debra Tomasino

**Affiliations:** 1 University of Connecticut, Waterbury, Connecticut, United States; 2 UConn, Mansfield, Connecticut, United States

## Abstract

This poster presentation highlights pilot findings of how older gay male drag queens define drag expression and its associated positive and negative attributes. While drag has become more mainstream, little is known about this sexual and gender minority (SGM) and what it means to be an older drag queen and how it interfaces with societal gender expectations. (Knutson, Koch, Sneed, Lee, & Chung, 2020; O’Brien, 2018). Research to date reports that while sexual minority youth experience bullying, anxiety, lower self-esteem, and suicidal ideation at higher rates than their heterosexual and gender conforming peers, research has not examined the role drag expression plays as a healthy coping mechanism and, in particular, what role it may play for older drag queens. (Levasseur, Kelvin, Rosskopf, 2013; Mueller, James, Abrutyn, & Levin, 2015). Several studies have found that familial support and connectedness offers valuable protective factors for sexual minority youth in their sexual identity development, but again, little is known about the benefits this may provide older drag queens (Brandon-Friedman & Kim, 2016; Eisenberg & Resnick, 2006). Utilizing Grounded Theory, in-depth interviews were conducted with gay males over the age of fifty (n=5) who identified as drag queens to understand how drag expression is integrated into one’s persona, how it may serve as a healthy coping mechanism, and how it interfaces with dragism, generativity, and family relationships over their lifespan (Donorfio, 2020). In addition to sharing the qualitative findings, demographic and data measures of personality, coping, resilience, and mood are also be reported.

